# Quality Matters More Than Quantity: Parent–Child Communication and Adolescents’ Academic Performance

**DOI:** 10.3389/fpsyg.2020.01203

**Published:** 2020-06-23

**Authors:** Yue Zhang

**Affiliations:** Psychology Department, Santa Clara University, Santa Clara, CA, United States

**Keywords:** parent–child communication, self-concept, academic performance, adolescents, indirect effect, structural equation modeling

## Abstract

This study examined the effects of parent–child communication, including the quantitative and qualitative aspects of these communications, on young American adolescents’ academic performance by using an Early Childhood Longitudinal Study-Kindergarten dataset. We considered children’s self-concept a mediator in examining the effects. Structural equation modeling was used for evaluating the model. The results revealed that the quality of parent–child communication was positively associated with these children’s academic performance, and this association can be mediated by the adolescents’ self-concept. However, the quantity of parent–child communication did not show a direct or an indirect association with the adolescents’ academic achievements. These findings suggest that efforts to improve the quality of parent–child communication and to strengthen the connection between high-quality communications and adolescents’ self-concept may benefit young adolescents’ academic development.

## Introduction

One of the biggest challenges in families with adolescents is parent–child communication. As children grow into adolescence, they developmentally desire independence and privacy (Grolnick et al., 2007; Hill and Tyson, 2009), and they are less likely to want to communicate with their parents about their school lives. Yet, parents may want to be informed about their children’s school lives, have frequent conversations with them, and remain helpful in their children’s educational experiences. Although parents would like to assist their children through these interactions, oftentimes they may overlook their children’s desire to be independent from their parents, especially during adolescence (Hill and Tyson, 2009). As a result, parent–child conflicts may increase and parents and their children may have less or even negative communications that involve quarrels and harsh conversations that might lead to adverse consequences, including poor school academic performance (Hay et al., 2016). Therefore, it is important to explore what parents should do in their daily struggles in communicating with their adolescents to support their academic performance.

Some studies have examined the association between the quantity of parent–child communication and children’s academic performance (Shochet et al., 2007; Park, 2008; Caro, 2011). In these studies, parent–child communication has been measured as the frequency of conversations between parents and their children about their school experiences in a given period of time (e.g., parents were asked, “How often do you talk with your child about his/her school progress, future plans, and homework?”). More frequent communications between parents and children on such issues appear to be associated with the children’s better academic performance (Shochet et al., 2007; Caro, 2011). Researchers supporting this association have explained that the quantity of parent–child communication on school-related issues is one of the crucial indicators of how much parents are involved in their children’s education. More frequent communications suggested a higher level of parental involvement and that the children’s academic performance might benefit from these parental practices. However, other researchers have claimed that more frequent conversations with children about their school lives may not be helpful in enhancing their academic achievement; instead, it may threaten these children’s needs for autonomy and may negatively influence their learning motivation (Keijsers and Poulin, 2013). When children are in their adolescent years, they tend to want independence from their parents and may not see the benefits or show any interest in conversing with their parents about their school-related issues (Grolnick et al., 2007; Hill and Tyson, 2009). Other studies have found that the quantity of parent–child communication does not significantly associate with children’s higher academic performance among middle and high schoolers (Jeynes, 2007; Park, 2008). Overall, studies primarily focusing on the quantity of parent–child communication indicated a mixed result in associating it with the children’s academic performance.

Besides the quantity of parent–child communication, another group of researchers has emphasized the significance of the quality aspect of these communications. Hollmann et al. (2016) described high-quality parent–child communication as parents and children conducting a conversation in an environment where the parents encourage their children’s opinions and provide structure with decisions that are tolerant of different viewpoints. In such a beneficial communication environment, both parents and children provide opinions and make decisions. The parents provide a structure with explanations of their actions and encourage their child or children to explain their opinions. In this way, the parents know the motivation behind and how to respond to their children’s actions and the children understand what and why something is expected of them. Studies on the quality aspect of parent–child communication have consistently suggested that parent–child communication involving parents’ understanding and support of their children is associated with the children’s better academic performance (Vukovic et al., 2013; Camacho-Thompson et al., 2016).

Although previous studies to date have documented the effect of the quantity or quality of parent–child communication on these children’s functioning in school, there has not been any single empirical research that has simultaneously investigated the relationship of the quantity and quality of parent–child communication to children’s academic performance among adolescents. Therefore, it is difficult to determine which aspects of communication parents need to pay more attention to—quantity or quality—to boost their adolescents’ academic performance. Hence, a few coherent suggestions from researchers have been generated for adolescents’ parents and family practitioners regarding the relative effects of parent–child quantity and quality communications about the children’s academic performance.

According to the social cognitive theory (Bandura, 1995), interactions between parents and their children can influence their self-evaluations of how capable they are in doing a given task. The cognitive abilities of individuals play a key role in their developmental outcomes in many aspects, including academic performance. A crucial component of social cognitive theory that has received much attention is *self-concept*, which consists of individuals’ judgments about their abilities and skills (Ahmed and Bruinsma, 2006). Research studies have demonstrated a direct association between children’s self-concept and their academic achievements (Wu and Kuo, 2015; Jhang, 2017; Susperreguy et al., 2017). On the other hand, individuals’ self-concept can be influenced by social agents, such as parents and teachers in the form of verbal persuasion. For example, parents can tell their children that they are capable of performing a task and will do it well. This effective method of verbal persuasion helps children realize that they can be successful in doing a task and that this success can translate to their new tasks (Urdan and Schoenfelder, 2006). More frequent positive communications between parents and children might be helpful in supporting the children’s positive self-concept, which contributes to their academic achievement (Rogers et al., 2009). However, few studies have investigated this mediation path of parent–child communication and the children’s academic performance via their self-concept. Even fewer studies have focused on the important quantity and quality aspects of parent–child communication. The role of children’s self-concept and how the different aspects of communication influence their academic performance is still not well understood. Therefore, this study considered the children’s self-concept as a mediator for examining what aspects of parent–child communication, quantity or quality, matter most to the children’s academic performance. In examining this association, we considered the potential confounding effect of the home environment on parent–child communication and children’s academic performance. Jeynes (2007) examined the effect of various forms of parental involvement, including parent–child communication, on the academic achievements of the students from grades 6 through 12. The researcher found that parent–child communication did not have a significant effect on the children’s academic achievement when their parents’ educational attainment was controlled. Consistent with Jeynes (2007), Park (2008) and Hay et al. (2016) stressed the necessity to control for family background characteristics, such as the parents’ educational attainment and family type, because they found that intact families with parents who had attained a higher education were more likely to have effective and healthy communications with their children and thus their academic achievement appeared to be higher than for children from divorced families or parents with lower educational attainment (Hao and Bonstead-Bruns, 1998; Wang et al., 2009).

## The Present Study

The objective of this current study was to investigate the associations between the different aspects of parent–child communication in terms of the quantity and the quality of those communications and the children’s academic performance. We also examined the possible indirect effect of parent–child communication on their academic performance via their self-concept. Because parent–child communication mostly occurs in a home environment, to avoid a possible confounding effect, we included parents’ educational attainment and family type as controls in our analysis because these factors may influence the way parents communicate with their children.

## Method

### Participants

This study’s findings are based on data from the Early Childhood Longitudinal Study-Kindergarten (ECLS-K, full sample). The ECLS-K followed 9000 children nationwide from kindergarten to the eighth grade. Our study focused on these eighth-graders in the ECLS-K dataset as our analysis sample because these children are transitioning from middle school to high school. To avoid a school-related confounding effect due to school changes, we selected eighth-graders who did not change schools during their first year of middle school. The participants in this study totaled 1815 eighth-graders who did not change schools during their middle school years (mean age = 14.31 years, SD = 0.47) and 52.6% were females. Among the participants, 1319 (72.7%) were White, 101 (5.6%) were African Americans, 231 (12.7%) were Hispanics, 77 (4.2%) were Asian American, and 87 (4.7%) were Others. The majority (89.9%) indicated that their native language is English, and most were in two-parent households plus siblings (69.4%). The parents had some college experience or a Bachelor’s degree (53.5% of the mothers and 40.5% of the fathers).

### Measures

#### Quantity of Parent–Child Communication

Four items assessing the frequency of parent–child conversations in a month were drawn from the parents’ questionnaire in the ECLS-K dataset. This communication content was about a child’s day at school, school friends, grades, and school activities. Ratings were measured on a four-point scale ranging from 1 (*not at all*) to 4 (*every day*). A higher score indicated a higher frequency of communication between parents and children. The one-factor confirmatory factor analysis model was acceptable for these four items (χ^2^ (2, *N* = 1815) = 23.18, *p <* 0.05, CFI = 0.99, RMSEA = 0.076, 90% CI [0.050, 0.105], SRMR = 0.020); standardized factor loadings were statistically significant, ranging from 0.54 to 0.82. The reliability of the four-item scale indicated a high internal consistency (Cronbach α = 0.76). Accordingly, we used the average score for the four quantities of parent–child communication items as the observed variable in the final structural equation model.

#### Quality of Parent–Child Communication

This communication involved parents’ understanding and support of their child or children in this study. We selected four items from the parents’ questionnaire in the ECLS-K dataset that reflected the supportive nature of parents in their conversations with their children. These items included aspects of how well parents get along with their children, make decisions together with them, understand, and trust them. Ratings were measured according to a four-point scale ranging from 1 (*never*) to 4 (*always*). Higher scores indicated a higher level for the quality of parent–child communication. The one-factor confirmatory factor analysis model was acceptable for these four items (χ^2^ (2, *N* = 1815) = 8.02, *p* < 0.05, CFI = 0.99, RMSEA = 0.041, 90% CI [0.014, 0.072], SRMR = 0.035); standardized factor loadings were statistically significant, ranging from 0.48 to 0.68. The reliability of the four-item scale indicated an acceptable internal consistency (Cronbach α = 0.60). Accordingly, we used the average score for the four quantities of parent–child communication items as the observed variable in the final structural equation model.

#### Academic Performance

Students’ eighth-grade academic performance in reading, math, and science were drawn from the ECLS-K dataset that assessed children’s academic progress in school in many ways, such as teachers’ and parents’ reports on the children’s school performance. However, these self-reported measures can be subjective and grades are likely to vary across schools, thus making it more difficult to determine the extent to which academic performance varies due to informants’ bias and schools and other factors. Taking this into consideration, we obtained the standardized test scores administrated by the ECLS-K researchers that assessed the students’ academic ability in reading, math, and science. These standard test scores were adjusted using the Item Response Theory (IRT) method to produce a more accurate estimate by the ECLS-K researchers (Najarian et al., 2009). The three-item measure of students’ academic performance indicated a high internal consistency (Cronbach α = 0.86).

#### Self-Concept

In the ECLS-K dataset, as part of the eighth-grade survey, the children provided information on their self-concept. They were asked about their level of agreement and the statements about certain perceptions about them. A total of seven items were included. Sample items included “I feel good about myself” and “I am able to do things as well as most of other people.” The children’s responses were based on a four-point Likert scale (1 = strongly disagree to 4 = strongly agree). The ECLS-K datafile included scores for each of these seven items that standardized to a mean of 0 and a standard deviation of 1. These seven items indicated high construct reliability (Cronbach α = 0.79). The one-factor confirmatory factor analysis model was acceptable for these seven items (χ^2^ (14, *N* = 1815) = 604.33, *p* < 0.05, CFI = 0.95, RMSEA = 0.051, 90% CI [0.042, 0.073], SRMR = 0.068); standardized factor loadings were statistically significant, ranging from 0.50 to 0.71. The ECLS-K researchers created the composite average score for this seven-item scale and used the composite indices as a measure of the children’s self-concept. Accordingly, we used the composite average score for the seven self-concept items as the observed variable in the final structural equation model. Higher score indicated a greater positive perception among the children about themselves.

#### Control Variables

We utilized parents’ educational attainment as measured from 1 (*below high school*) to 6 (*doctorate degree*) as a control variable. We also used parents’ reporting of their current marital status (married, divorced, or separated) as an indicator of family type as another control variable. For analytical purposes, a dummy variable was created to represent incomplete or intact families by recoding this variable as married (0 = other status, 1 = yes).

### Analytical Strategy

We used structural equation modeling (SEM) in AMOS 20.0 (Arbuckle, 2011) in three steps to test the associations between the quantity and quality of parent–child communication and the children’s academic performance via their self-concept. First, we conducted a correlation matrix to identify the initial association between these aspects of parent–child communication, children’s self-concept, and academic performance. Second, the fit of the hypothesized model was evaluated using three criteria: a ratio of χ^2^ to df (χ^2^*/df*) < 5 (Wheaton et al., 1977); comparative fit index (CFI) > 0.90 (Bentler, 1990); and root mean square error of approximation (RMSEA) < 0.08 with 90% CI (Schreiber et al., 2006), and standardized root mean square residual (SRMR) < 0.08 (Hu and Bentler, 1999). We also examined the association between parent–child communication, children’s self-concept, and their academic performance, which accounted for the controls in our study. As for missing data, full information maximum likelihood estimation was used (Acock, 2005). Lastly, we used the bootstrapping method with bias-corrected 95% confidence intervals to examine the indirect effect of the quantity and quality of parent–child communication on the children’s academic performance through their self-concept.

## Results

Table 1 presents the descriptive statistics and correlation matrix among the variables. The quantity (*M* = 3.59, SD = 0.43, range = 4) and quality of parent–child communication (*M* = 3.36, SD = 0.46, range = 4) indicated a moderate to high level of communications in terms of quantity and quality occurred between parents and children. Children’s self-concept indicated an average level of their perception of themselves (*M* = 0.06, SD = 0.85). As shown in Table 1, the quantity of parent–child communication has a negative association with the children’s academic performance in different subject areas (*r_reading_* = −0.01, *p >* 0.05, *r_math_* = −0.02, *p* < 0.05, *r*_*science*_ = −0.03, *p* < 0.01), whereas the quality of parent–child communication indicated a significant positive association with children’s academic performance (*r_reading_* = 0.06, *r_math_* = 0.07, *r*_*science*_ = 0.04, *p*s < 0.01). The children’s self-concept had a non-significant association with the quantity of parent–child communication (*r* = 0.02, *p* > 0.05) but had a significant and positive association with the quality of parent–child communication (*r* = 0.06, *p* < 0.01).

**TABLE 1:** Parent–child communication variables, self-concept, and academic performance variables: correlations and descriptive statistics (*N* = 1815).

Variables	1	2	3	4	5	6
1. Quantity of parent–child communication	–					
2. Quality of parent–child communication	0.20**	–				
3. Self-concept	0.02	0.06**	–			
4. Reading score	-0.01	0.06**	0.16**	–		
5. Math score	-0.02*	0.07**	0.15**	0.65**	–	
6. Science score	-0.03**	0.04**	0.15**	0.69**	0.73**	–
*M*	3.59	3.36	0.06	172.73	144.23	86.34
SD	0.43	0.46	0.85	29.63	21.54	14.9

*p < 0.05, **p < 0.01.

**FIGURE 1:**
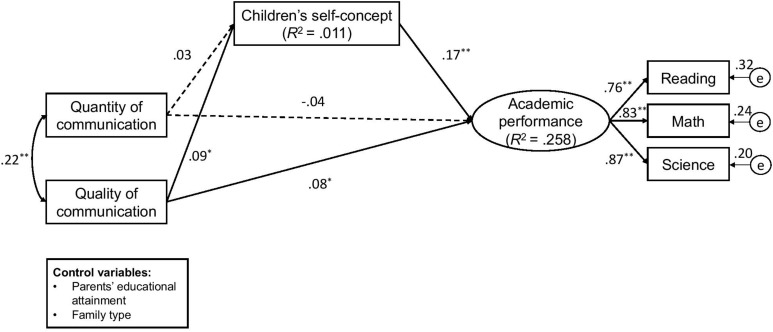
Standardized coefficients of the effects of parent-child communication on the latter’s academic performance via their self-concept. Solid lines are the significant paths (^∗^*p* < 0.05, ^∗∗^*p* < 0.01) and dashes are the non-significant paths. Model fit: χ^2^ (12, *N* = 1815) = 15.24, χ^2^/*df* = 1.270, *p* = 0.228 > 0.05, CFI = 0.999, RMSEA = 0.012, 90% CI [0.000, 0.028], SRMR = 0.007. Parents’ educational attainment and family type (1 = married, 0 = other status) were control variables in the analysis.

Next, we tested the hypothesized model while including control variables for the study. The data showed a satisfactory model fit, χ^2^ (12, *N* = 1815) = 15.24, χ^2^/*df* = 1.270, *p* = 0.228 > 0.05, CFI = 0.999, RMSEA = 0.012, 90% CI [0.000, 0.028], SRMR = 0.007. The model explained 25.8% of the variance in children’s academic performance. We examined the associations between the quantity and quality of parent–child communication with the children’s academic performance. Figure 1 presents the standardized coefficients in the effects of parent–child communication on the children’s academic performance via their self-concept. The quality of parent–child communication indicated a positive association with their children’s academic performance (β = 0.079, *p* < 0.05). However, the quantity of parent–child communication did not significantly associate with the latter’s academic performance (β = −0.042, *p* > 0.05; see Figure 1). These findings indicated that a high quality of parent–child communication had a positive effect on the latter’s academic performance while the quantity of communication did not show any effect. For the control variables, we found that parents’ educational attainment was significantly correlated with children’s self-concept (β = 0.060, *p* < 0.05) and academic achievement (β = 0.250, *p* < 0.01), whereas family type was not significantly associated with children’s self-concept (β = −0.006, *p* > 0.05) or academic achievement (β = −0.004, *p* > 0.05).

Lastly, we evaluated the indirect effects of the quantity and quality of parent–child communication on the latter’s academic performance through their self-concept. Table 2 presents the indirect effect of parent–child communication on the children’s academic performance via their self-concept. The quality of parent–child communication indicated a significant indirect effect on the children’s academic performance through their self-concept (β = 0.007, 95% CI [0.002, 0.013]), while the quantity of parent–child communication did not reveal such an indirect effect through the children’s self-concept (β = −0.002, 95% CI [−0.007, 0.004]; see Table 2).

**TABLE 2:** Indirect effect of parent–child communication on children’s academic performance.

Communication dimensions	Effect of communication dimensions on self-concept	Effect of self-concept on academic performance	Indirect effect of self-concept	Bootstrapped 95% CI for indirect effect
Quantity of parent–child communication	0.031	0.174**	−0.002	[−0.007, 0.004]
Quality of parent–child communication	0.088*	0.174**	0.007*	[0.002, 0.013]

CI, confidence interval, *p < 0.05, **p < 0.01, two tailed.

## Discussion

This study examined how the quantity and quality of parent–child communication were associated with adolescents’ academic performance through the children’s self-concept. We found that high-quality parent–child communication had a significantly positive effect on children’s academic performance. This effect can be mediated by the children’s self-concept. However, the quantity of parent–child communication did not have an effect on the children’s self-concept or academic performance.

These findings suggest that the quality of parent–child communication may show a closer connection with these children’s academic performance than the quantity of communication. Parents’ having more frequent conversations with their children about their school lives may not be helpful in enhancing these adolescents’ academic performance. This may be because children in their adolescent years tend to want independence from their parents (Grolnick et al., 2007; Hill and Tyson, 2009) and may not see the benefits or show any interest in conversing with their parents; instead, they find such attempts to have frequent conversations about school life means giving away their privacy, power, and identity (Solomon et al., 2002). However, the alternative aspect of parent–child communication—the quality of such communications—does contribute to children’s academic enhancement. Specifically, if parents would show their trust and understanding of their child during these conversations and would like to collaborate with them in decision making, then these adolescents’ academic performance is likely to benefit from such high-quality communications with their parents.

We also found that high-quality, parent–child communication benefit adolescents’ academic performance by influencing their positive self-concept. This finding supports Bandura’s social cognitive theory and is consistent with a prior study (Rogers et al., 2009) showing that positive parent–child interactions (i.e., mothers’ academic encouragement) had an indirect effect on children’s academic performance through their self-concept. In high-quality communications during which parents listen to and respect their children’s perspectives, invite them to be part of the decision-making process, understand their emotions, and provide them with conducive feedback; these children are more likely to actively participate in family conversations and express their perspectives when facing critical school-related choices (e.g., courses, study plans). Through such parent–child communication, parents socialize them into being autonomous, confident in their decisions, and tolerant of others’ perspectives. Thus, such collaborative interactions support the children’s needs for autonomy and the establishment of a positive self-perception, which is especially important when they are in their adolescent years (Hill and Tyson, 2009). When children are confident about themselves, they tend to perform better in what they do and they are more likely to have high academic achievements in school (Guay et al., 2013; Hollmann et al., 2016). The findings of our research emphasized that high-quality, parent–child communication can promote children’s academic performance through their self-concept.

The current study is subject to several limitations. The cross-sectional design limited conclusions about causes and effects. Some scales, such as the quality of parent–child communication, displayed somewhat low reliability. Despite these limitations, the present study examines the quality and quantity of parent–child communication as it relates to the latter’s academic performance and allows us to identify which aspects of parent–child communication (quantity vs. quality) are more influential in adolescents’ academic performance. In conclusion, frequent parent–child communication about their children’s school issues appears to be less influential than the quality of communication about the children’s academic performance. This study argued that what matters more to young adolescents’ academic performance is the quality of their parent–child communication rather than the quantity. These findings shed light on how parents and educational programs can advance efforts in helping adolescents to enhance their academic performance.

## Data Availability Statement

The datasets generated for this study are available on request to the corresponding author.

## Ethics Statement

Ethical review and approval was not required for the study on human participants in accordance with the local legislation and institutional requirements. Written informed consent from the participants’ legal guardian/next of kin was not required to participate in this study in accordance with the national legislation and the institutional requirements.

## Author Contributions

The author contributed to this study in aspects of manuscript writing, data analysis, research design, and editing. The author confirms being the sole contributor of this work and has approved it for publication.

## Conflict of Interest

The author declares that the research was conducted in the absence of any commercial or financial relationships that could be construed as a potential conflict of interest.

## Appendix

**Table:** List of items used for measuring the quantity and quality of parent–child communication, self-concept, and academic performance (Najarian et al., 2009).

Construct	Description	Items in the ECLS-K dataset	Scale of measurement
Quantity of parent–child communication	Four items from parents’ report assessing the frequency of parent–child conversations in a month	1. P7OFTTLK: “How often do you talk with your child about his/her day at school?” 2. P7TLKFRD: “How often do you talk with your child about his/her friends? 3. P7TLKGRD: “How often do you talk with your child about his/her grades?” 4. P7TLKSCH: “How often do you talk with your child about his/her school activities?”	1 = not at all, 4 = every day
Quality of parent–child communication	Four items from parents’ report that reflected the supportive nature of parents in their conversations with their children	1. P7GETALN: “You get along well with your child.” 2. P7DECIDE: “You and your child make decisions about his/her life together.” 3. P7NOUNDR: “You just do not understand him/her.” (recode) 4. P7TRUST: “You feel you can really trust him/her.”	1 = never, 4 = always
Children’s self-concept	Seven items from children’s report on their level of agreement on certain perceptions about themselves	1. C7FLGOOD: “I feel good about myself.” 2. C7WORTH: “I feel I am a person of worth, the equal of other people.” 3. C7ABLE: “I am able to do things as well as most other people.” 4. C7SATISF: “On the whole, I am satisfied with myself.” 5. C7USELES: “I certainly feel useless at times.” (recode) 6. C7NOPRD: “I feel I do not have much to be proud of.” (recode) 7. C7NOGOOD: “At times I think I am no good at all.” (recode)	1 = strongly disagree, 4 = strongly agree
Academic performance	Standardized test scores administrated by the ECLS-K researchers that assessed the students’ academic ability in reading, math, and science, collected during the spring of the student’s eighth-grade year	1. C7R4RSCL: reading IRT scale score. 2. C7R4MSCL: math IRT scale score. 3. C7R2SSCL: science IRT scale score.	–
